# Molecular, structural and biochemical characterization of a novel recombinant chlorophyllase from cyanobacterium *Oscillatoria acuminata* PCC 6304

**DOI:** 10.1186/s12934-020-01507-w

**Published:** 2021-01-12

**Authors:** Sitian Gu, Xiaojun Dai, Zhengjun Xu, Qiwen Niu, Jiang Jiang, Yuanfa Liu

**Affiliations:** 1 State Key Laboratory of Food Science and Technology, Collaborative Innovation Center of Food Safety and Quality Control in Jiangsu Province, National Engineering Research Center for Functional Food, National Engineering Laboratory for Cereal Fermentation Technology, School of Food Science and Technology, Jiangnan University, 1800 Lihu Road, Jiangsu 214122 Wuxi, People’s Republic of China; 2 Wilmar Biotechnology Research & Development Center Co., Ltd, 200137 Shanghai, People’s Republic of China

**Keywords:** Chlorophyllase, *Oscillatoria acuminata* PCC 6304, Biochemical characteristic, Substrate specificity, Catalytic triad

## Abstract

**Background:**

Chlorophyllase catalyzes the hydrolysis of chlorophyll and produces chlorophyllide and phytol. Cyanobacterial chlorophyllases are likely to be more highly heterologously expressed than plant chlorophyllases. A novel recombinant chlorophyllase from the cyanobacterium *Oscillatoria acuminata* PCC 6304 was successfully expressed in *Escherichia coli* BL21(DE3).

**Results:**

The putative N-terminal 28-amino-acid signal peptide sequence of *O. acuminata* chlorophyllase (OaCLH) is essential for its activity, but may confer poor solubility on OaCLH. The C-terminal fusion of a 6 × His tag caused a partial loss of activity in recombinant OaCLH, but an N-terminal 6 × His tag did not destroy its activity. The optimal pH and temperature for recombinant OaCLH activity are 7.0 and 40 °C, respectively. Recombinant OaCLH has hydrolysis activities against chlorophyll a, chlorophyll b, bacteriochlorophyll a, and pheophytin a, but prefers chlorophyll b and chlorophyll a as substrates. The results of site-directed mutagenesis experiments indicated that the catalytic triad of OaCLH consists of Ser159, Asp226, and His258.

**Conclusions:**

The high-level expression and broad substrate specificity of recombinant OaCLH make it suitable for genetically engineering and a promising biocatalyst for industrial production, with applications in vegetable oil refining and laundry detergents.

## Background

Chlorophyll (Chl) is the commonest pigment in nature, occurring widely in plants, algae, and cyanobacteria. Chlorophyllase (Chlase, EC 3.1.1.14) is one of the most important enzyme in the chlorophyll metabolism of all photosynthetic organisms. It catalyzes the hydrolysis of chlorophyll, producing chlorophyllide (Chlide) and phytol [1].

The catalytic activity of chlorophyllase has potential industrial and pharmaceutical applications. It can remove green pigments from edible oils to improve their oxidative stability, and can replace the expensive adsorptive bleaching technique currently used in the edible oil refining industry [2]. The reaction products of chlorophyllase, chlorophyllides and their derivatives, have been shown to have antiviral, antioxidant, antimutagenic, and anticarcinogenic activities in vitro [3–7].

Chlorophyllase activity was first discovered in 1913, and many studies have investigated the physiological and biochemical properties of the chlorophyllases partially or completely purified from various plant and algal species, including *Chenopodium album* [8], *Citrus limon* [9], *Chlorella regularis* [10], and *Phaeodactylum tricornutum* [11, 12]. Chlorophyllase is considered a membrane-bound protein that normally localizes in chloroplasts and the thylakoid membrane [13]. Okazaea et al. demonstrated that the recombinant chlorophyllase from *Ginkgo biloba* localizes in the thylakoid membranes of the chloroplast [14]. Shemer et al. showed that chlorophyllase from *Citrus limon*, which is induced by ethylene, localizes in the plastid [9]. However, two chlorophyllases from *Arabidopsis thaliana* are reported to localize outside the plastid, in the endoplasmic reticulum and tonoplast [15, 16].

Many recombinant plant and algal chlorophyllases, including those from *Chenopodium album* [17], *Triticum aestivum* [18], *Pachira macrocarpa* [19], *Citrus sinensis* [20], *Brassica oleracea* [21], and *Chlamydomonas reinhardtii* [22], have been expressed in the *Escherichia coli* system and characterized biochemically. However, the heterologous expression of recombinant plant chlorophyllases is usually very low. When Arkus et al. expressed the chlorophyllase of *Triticum aestivum* (wheat) in *E. coli*, the recombinant target protein content in the growth medium was only ~ 5 mg/L [18]. Therefore, plant chlorophyllases are generally difficult to use in industrial applications.

Microbial enzymes are likely to be more highly heterologously expressed than plant enzymes. Like the plant and algal chlorophyllases, the cyanobacterial chlorophyllases play an important role in chlorophyll metabolism of cyanobacteria.

Only a few studies of cyanobacterial chlorophyllase have been reported. Chou et al. isolated and expressed a chlorophyllase from *Cyanothece* sp. ATCC 51142 in *E. coli*, which preferentially hydrolyzed bacteriochlorophyll a (BChl a) to bacteriochlorophyllide a (BChlide a) and phytol [23]. However, the physiological function of the cyanobacterial chlorophyllases has not been investigated.

*Oscillatoria acuminata* PCC 6304 is a photosynthetic filamentous cyanobacterium. Its complete chromosomal genomic sequence was fully determined and published in GenBank (NCBI database) in 2012. The putative chlorophyllase sequence was annotated, but has not been investigated. In this study, the chlorophyllase-related gene (GeneID: 428001063) of *O. acuminata* PCC 6304 was synthesized and expressed in an *E. coli* system. The molecular structure, substrate specificity, enzyme kinetics, and biochemical characterization of recombinant *O. acuminata* chlorophyllase (OaCLH) were then investigated.

## Results and discussion

### Sequence analysis of recombinant OaCLH

The nucleotide sequence of *OaCLH* contains 978 base pairs, encoding 325 amino acid residues with a calculated molecular mass of 34.78 kDa and a predicted pI of 4.55. The TargetP-2.0 server and SignalP-5.0 server predicted the presence of a putative 28-amino-acid signal peptide at the N-terminal end of the OaCLH sequence. The signal peptide presumably guides OaCLH to the thylakoid membrane, in which the chlorophylls and other photosynthetic pigments of cyanobacteria *O. acuminata* PCC 6304 localize. Various physical and chemical parameters of OaCLH, predicted with the ProtParam tool, are shown in Table 1. The estimated half-life of OaCLH is 30 h in vitro in mammalian reticulocytes, more than 20 h in vivo in yeast, and more than 10 h in vivo in *E. coli*. Its instability index was computed to be 37.42, which classifies OaCLH as stable. The calculated aliphatic index (101.14) indicates that OaCLH is thermostable. However, the positive grand average hydropathy (GRAVY) value (0.157) indicates that OaCLH is a poorly hydrophilic protein, which may confer poor solubility. The TMpred server predicted four potential transmembrane domains in the OaCLH sequence, so OaCLH may be a membrane-bound protein of *O. acuminata* PCC 6304.

**Table 1:** Various physical and chemical parameters of OaCLH predicted with the ProtParam tool

Source	Accession no.	Molecular weight	pI	Estimated half-life (hours)			Instability index	Aliphatic index	GRAVY
				mammalian reticulocytes, in vitro	yeast, in vivo	*E. coli*, in vivo			
*Oscillatoria acuminata*	AFY81906.1	34783.41	4.55	30	> 20	> 10	37.42	101.14	0.157

**Fig. 1:**
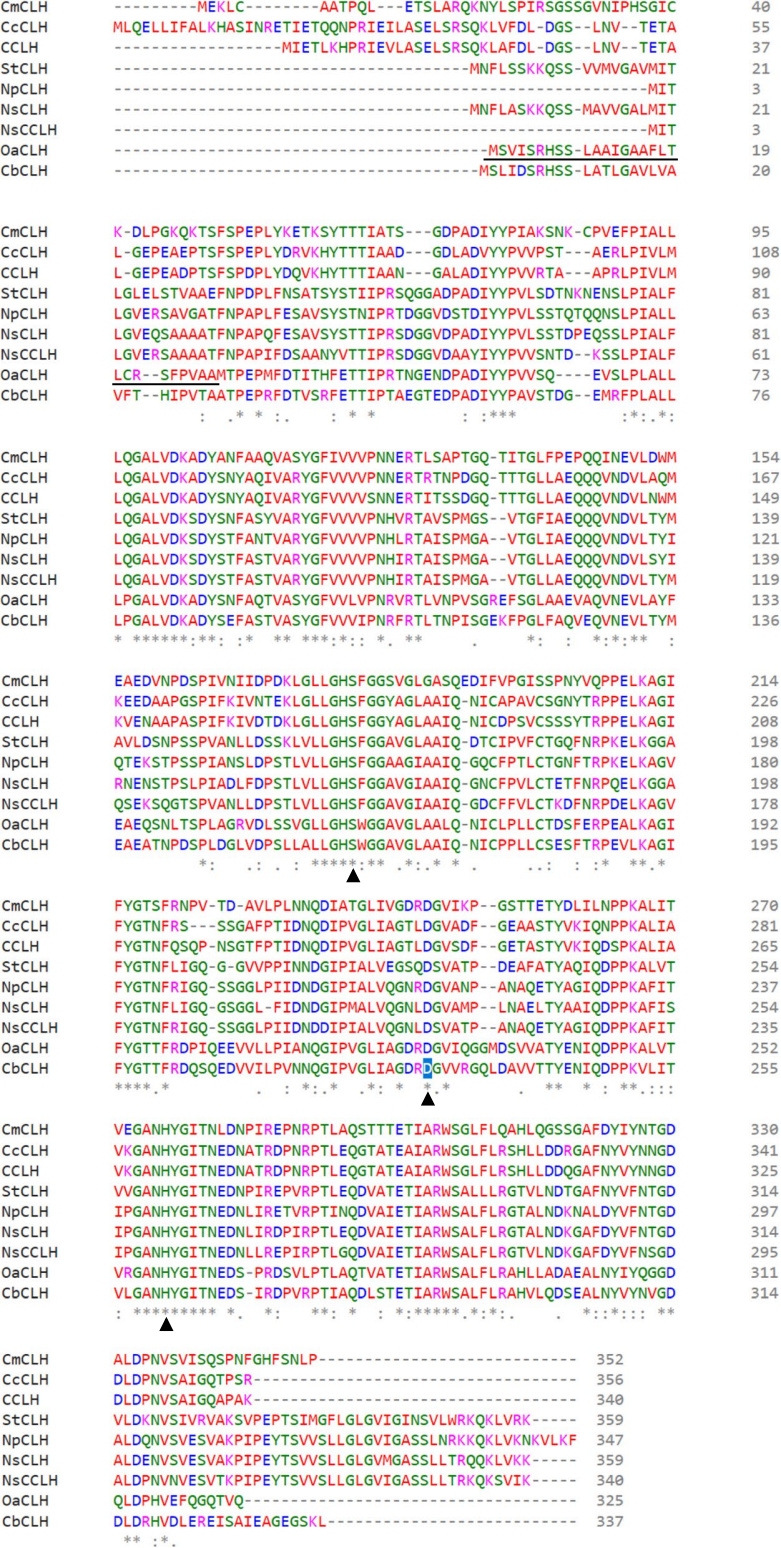
Multiple amino acid sequence alignment of various cyanobacteria chlorophyllases. These cyanobacteria chlorophyllases are from *Oscillatoria acuminata* PCC 6304, *Nostoc* sp. PCC 7524, *Scytonema tolypothrichoides* VB-61278, *Chamaesiphon minutus* PCC 6605, Cyanobacteria bacterium J007, *Chroococcidiopsis cubana* CCALA 043, cyanobacterium TDX16, *Nostoc punctiforme* NIES-2108, and *Nostoc* sp. CENA543. Asterisks, colons, and periods represent identity, strong similarity, and weak similarity, respectively. Three black triangles denote the putative catalytic triad: Ser, Asp, and His residues. The predicted signal peptide sequence of OaCLH is underlined

Recombinant OaCLH shares a higher degree of amino acid sequence identity with other cyanobacterial chlorophyllases (51–71%) than with plant chlorophyllases (25–32%). Clustal Omega was used to align the amino acid sequence of OaCLH with eight previously reported cyanobacterial chlorophyllase amino acid sequences: from *Nostoc* sp. PCC 7524, *Scytonema tolypothrichoides* VB-61278, *Chamaesiphon minutus* PCC 6605, Cyanobacteria bacterium J007, *Chroococcidiopsis cubana* CCALA 043, cyanobacterium TDX16, *Nostoc punctiforme* NIES-2108, and *Nostoc* sp. CENA543. The multiple sequence alignment (Fig. 1) showed that all these cyanobacterial chlorophyllases share a highly conserved sequence (GHSXG), which is also present in all plant chlorophyllases [24]. Specifically, this conserved motif is GHSRG in plant chlorophyllases, but GHSFG in cyanobacterial chlorophyllases other than OaCLH and chlorophyllase from Cyanobacteria bacterium J007, which contain a GHSWG motif. This conserved motif belongs to all α/β hydrolase family domains (pfam12695), which are reported to have several functions and activities similar to those of proteases, lipases, peroxidases, esterases, epoxide hydrolases, and dehalogenases [25]. This conserved motif contains a serine residue, which is responsible for the nucleophilic attack on the carbonyl carbon atom of the ester bond in the hydrolysis reaction. Therefore, the presence of the conserved motif GHSXG underpins the hydrolysis activity of the chlorophyllases.

The multiple sequence alignment also showed that the putative catalytic residues (serine [Ser]–aspartic acid [Asp]–histidine [His]) are strictly conserved in all cyanobacterial chlorophyllases (marked with three black triangles in Fig. 1). The putative catalytic triad of OaCLH may be Ser159, Asp226, and His258.

**Fig. 2:**
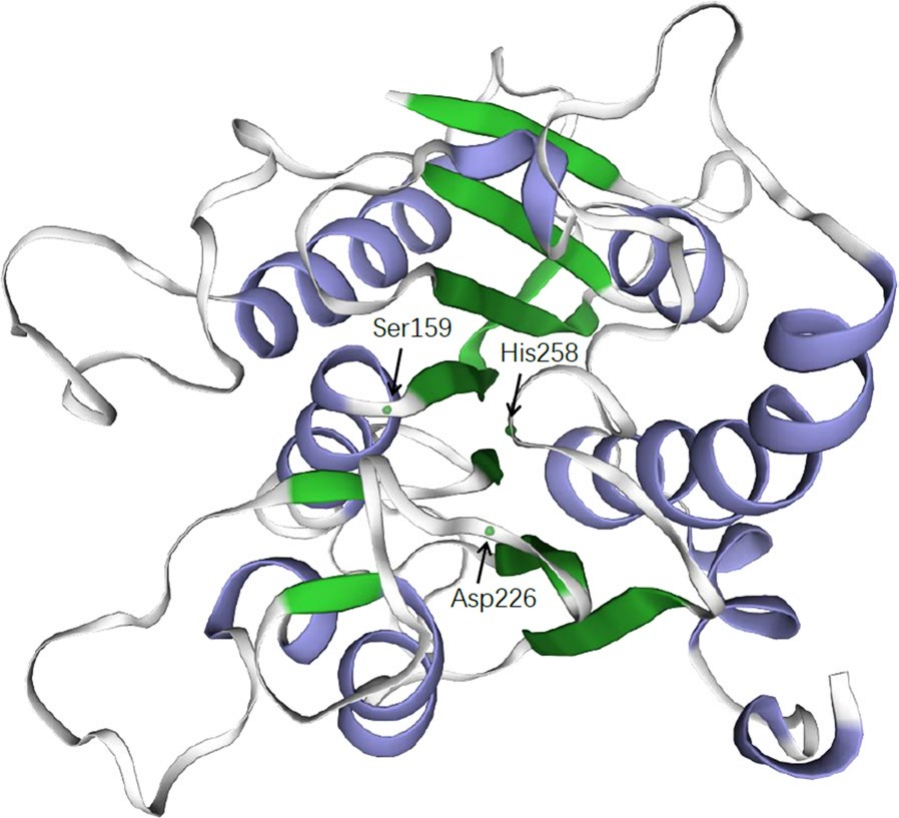
Predicted tertiary structure of OaCLH. This diagram was constructed with the SWISS-MODEL server. The conserved catalytic triad residues (Ser159, Asp226, and His258) of OaCLH are indicated

The predicted tertiary structural model of OaCLH (Fig. 2) was constructed with the SWISS-MODEL program using a template (6scd.1.A) of polyester hydrolase PE-H from *Pseudomonas aestusnigri*, which is the closest homologue of OaCLH [26]. OaCLH shares 21.30% identity with the template 6scd.1.A, based on the overall amino acid sequence. The tertiary structural model is composed of seven β-strands and nine α-helices. Active serine residue 159 is located in an extremely sharp turn between a β-strand and an α-helix, called the ‘nucleophilic elbow’, a conserved structure in the α/β hydrolases. However, the predicted structural model lacks the active site lid that is a common feature of the α/β hydrolase family and usually lies over the active site. The putative catalytic triad (Ser159, Asp226, and His258) is located on a surface-exposed loop and clusters as the catalytic domain.

### Expression and purification of recombinant OaCLH

**Fig. 3:**
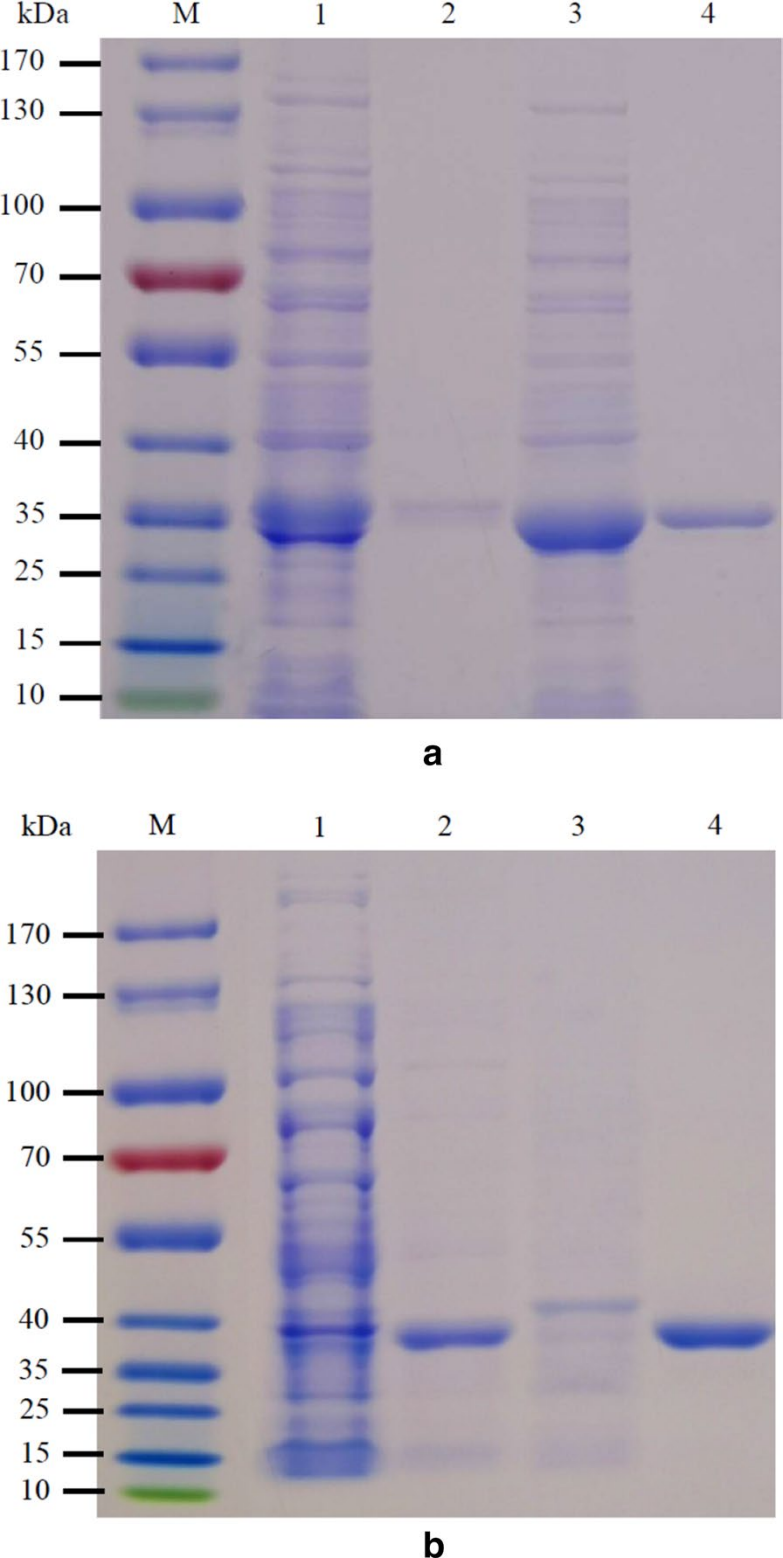
**a** SDS-PAGE analysis of C-terminally 6 × His-tagged recombinant OaCLHs. M, prestained molecular weight marker. Lane 1, crude extract of recombinant full-length OaCLH. Lane 2, purified recombinant full-length OaCLH after Ni^2+^ affinity chromatography. Lane 3, crude extract of recombinant truncated OaCLH. Lane 4, purified recombinant truncated OaCLH after Ni^2+^ affinity chromatography. The protein gel was stained with Coomassie Brilliant Blue **b** SDS-PAGE analysis of N-terminally 6 × His-tagged recombinant OaCLHs. M, prestained molecular weight marker. Lane 1, crude extract of recombinant full-length OaCLH. Lane 2, crude extract of recombinant truncated OaCLH. Lane 3, purified recombinant full-length OaCLH after Ni^2+^ affinity chromatography. Lane 4, purified recombinant truncated OaCLH after Ni^2+^ affinity chromatography. The protein gel was stained with Coomassie Brilliant Blue

The full-length and signal-peptide-deleted nucleotide sequences of the *OaCLH* gene were synthesized and cloned into the pET-24a(+) and pET-28a(+) vectors, respectively. *Escherichia coli* BL21(DE3) cells were transformed with the corrected recombinant expression plasmids to overexpress the genes under induction with isopropyl β-D-1-thiogalactopyranoside (IPTG). The soluble recombinant OaCLHs fused with a C-terminal or N-terminal 6 × His tag was purified with Ni^2+^ affinity chromatography. The expression of the full-length recombinant OaCLH reached ~ 150 mg/L in growth medium, significantly higher than the yield (5 mg/L) of plant chlorophyllase from *Triticum aestivum* expressed in *E. coli* [16], which is the typical level of plant chlorophyllase expression. Therefore, compared with plant chlorophyllases, recombinant OaCLH is significantly more strongly expressed in *E. coli*. SDS-PAGE (Fig. 3) showed that the molecular weights of the full-length and truncated OaCLHs corresponded to the calculated molecular masses of the 6 × His-tagged recombinant OaCLHs. SDS-PAGE also demonstrated that the expression level of soluble full-length OaCLH was significantly lower than that of truncated OaCLH. This is attributed to the poor solubility of full-length OaCLH conferred by the putative 28-amino-acid signal peptide.

**Fig. 4:**
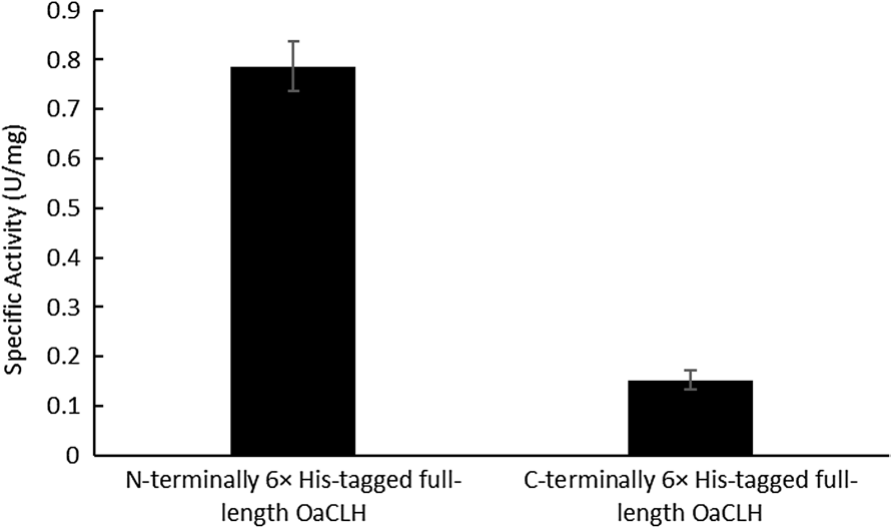
Effect of N-terminal and C-terminal fusion with a 6 × His tag on the activity of recombinant full-length OaCLH. Specific activities of purified N-terminally and C-terminally 6 × His-tagged full-length OaCLH were measured with the standard chlorophyllase activity assay method, using Chl a as the substrate. The Bradford method was used to quantify the protein. Values are the means ± standard deviation (SD) of three independent experiments

The specific activities of the purified C-terminally and N-terminally 6 × His-tagged full-length OaCLHs were measured. The results (Fig. 4) showed that the specific activity of C-terminally 6 × His-tagged OaCLH was significantly lower than that of N-terminally 6 × His-tagged OaCLH. A multiple sequence alignment and predicted tertiary structural model showed that the putative catalytic active center of OaCLH is located at the C-terminus. Therefore, C-terminal fusion with a 6 × His tag is likely to change the conformation of the active center, leading to a partial loss of chlorophyllase activity. Furthermore, when N-terminally 6 × His-tagged OaCLH was digested with thrombin to remove its 6 × His tag, the specific activity of the resulting OaCLH did not differ significantly from that of the 6 × His-tagged protein (data not shown). Therefore, the N-terminal 6 × His tag does not destroy the activity of OaCLH.

**Fig. 5:**
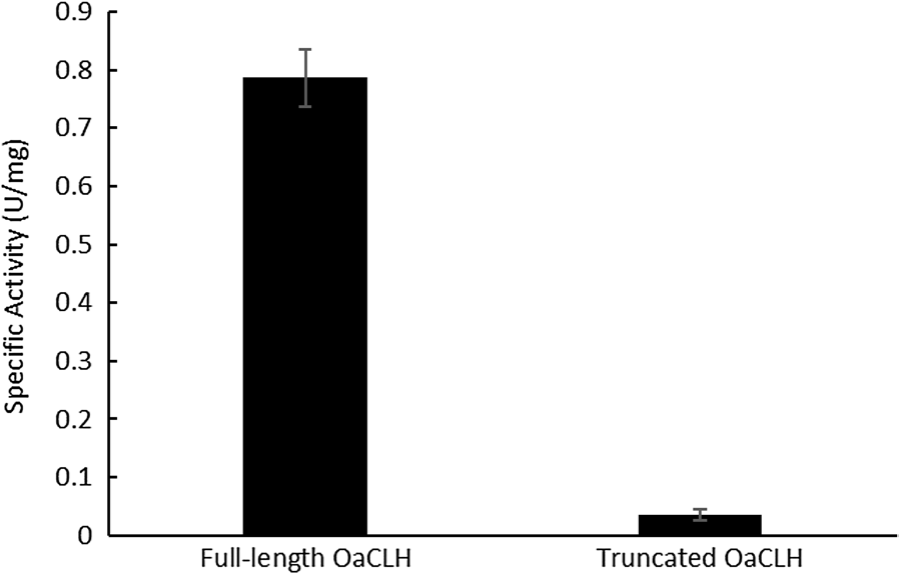
Effect of the predicted N-terminal signal peptide on the activity of recombinant OaCLH. Specific activities of purified full-length and truncated OaCLHs were measured with the standard chlorophyllase activity assay method, using Chl a as the substrate. The Bradford method was used to quantify the protein. Values are the means ± SD of three independent experiments

Previous studies have shown that the N-terminal transit peptide of chlorophyllase plays an important role in its Chl hydrolysis activity. Chen et al. reported that the chlorophyllases from *Pachira macrocarpa* from which the N-terminal transit peptide was removed were functionally inactive [19]. However, Harpaz-Saad et al. showed that the removal of the N-terminal 21 amino acids of chlorophyllase from *Citrus sinensis* generated a more active enzyme in vivo [27]. The presence of a putative 28-amino-acid signal peptide was predicted at the N-terminal end of the OaCLH sequence. The TMpred server also predicted that the amino acid sequence from Leu 10 to Met 29 is an N-terminally located transmembrane domain, which could anchor OaCLH within the thylakoid membrane, in which the cyanobacterial chlorophylls localize. To evaluate the role of the predicted N-terminal signal peptide in the activity of recombinant OaCLH, an in vitro chlorophyllase activity assay was performed using purified full-length and N-terminally truncated OaCLHs and Chl a as the substrate. The results (Fig. 5) indicated that full-length OaCLH displayed significantly higher specific chlorophyllase activity than truncated OaCLH. The removal of the predicted signal peptide sequence caused more than 95% activity loss in OaCLH. Therefore, the predicted N-terminal signal peptide sequence of recombinant OaCLH is essential for its activity.

The biochemical characterization and kinetics of purified N-terminally 6 × His-tagged full-length OaCLH were investigated in this work.

### Biochemical characterization of recombinant OaCLH

**Fig. 6:**
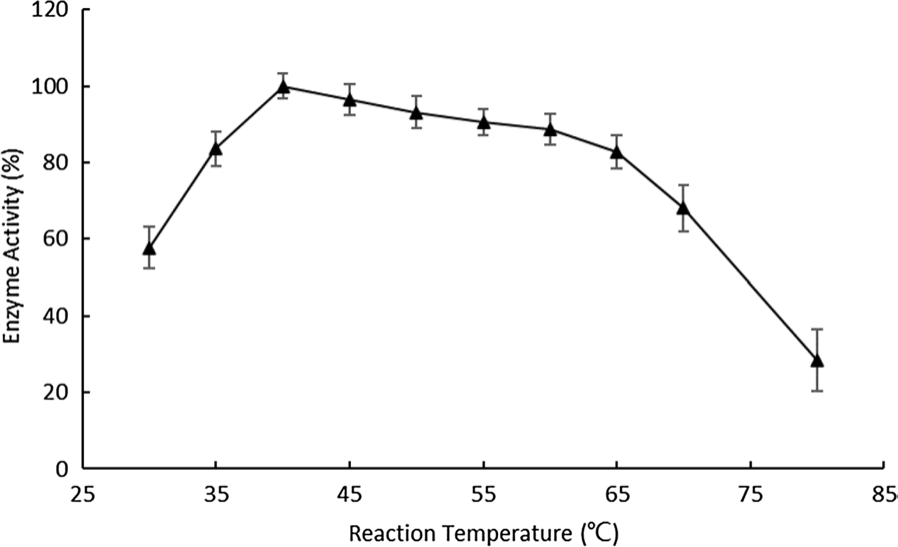
Effect of temperature on the activity of recombinant OaCLH. The optimal temperature of recombinant OaCLH was measured with the standard chlorophyllase activity assay method, using Chl a as the substrate. Values are the means ± SD of three independent experiments

The recombinant OaCLH had an optimal temperature for Chl a hydrolysis of 40 °C, and maintained > 80% of its peak activity in a temperature range of 35–65 °C. However, more than 70% of its chlorophyllase activity was lost when the reaction temperature reached 80 °C (Fig. 6). Khalyfa et al. reported that the optimum temperature range for chlorophyllase activity is 25–37 °C, and the optimum reaction temperature for *Pheaodactylumn tricornutum* chlorophyllase is 31 °C [12]. Chou et al. demonstrated that the optimum temperatures for the catalytic activities of chlorophyllase 1 from *Chlamydomonas reinhardtii* and chlorophyllase from *Cyanothece* sp. ATCC 51142 were 40 °C and 60 °C, respectively [22, 23].

**Fig. 7:**
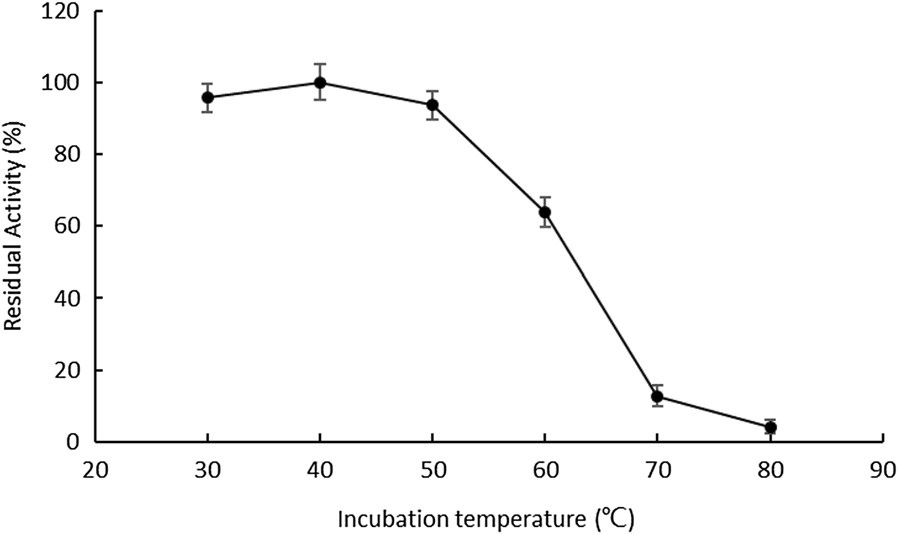
Effect of temperature on the thermal stability of recombinant OaCLH. The residual activities of recombinant OaCLH were measured with the standard chlorophyllase activity assay method, using Chl a as the substrate. Values are the means ± SD of three independent experiments

The recombinant OaCLH remained stable at 30, 40, and 50 °C. After incubation for 60 min at 30, 40, or 50 °C, more than 90% of its catalytic activity was retained. When the incubation temperature increased to 60 °C, the residual activity was approximately 64% after incubation for 60 min. After incubation for 60 min at 70 °C and 80 °C, only 13% and 4% of its activity remained, respectively (Fig. 7).

**Fig. 8:**
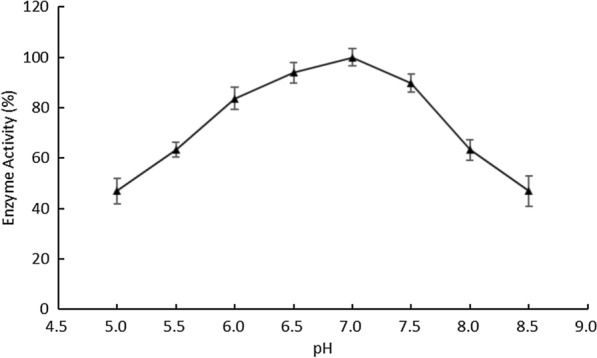
Effect of pH on the activity of recombinant OaCLH. The optimal pH of recombinant OaCLH was measured with the standard chlorophyllase activity assay method, using Chl a as the substrate. Values are the means ± SD of three independent experiments

The optimal pH of the recombinant OaCLH for Chl a hydrolysis is 7.0, and about 90% of its peak activity was detected at pH 6.5 and pH 7.5 (Fig. 8). These results agree well with the fact that most chlorophyllases from various sources have optimal pHs close to neutrality, as previously reported by Tsuchiya et al. [8]. Chou et al. also reported that the optimal catalytic activity of chlorophyllase from *Cyanothece* sp. ATCC 51142 occurred at pH 7.0 [23].

**Fig. 9:**
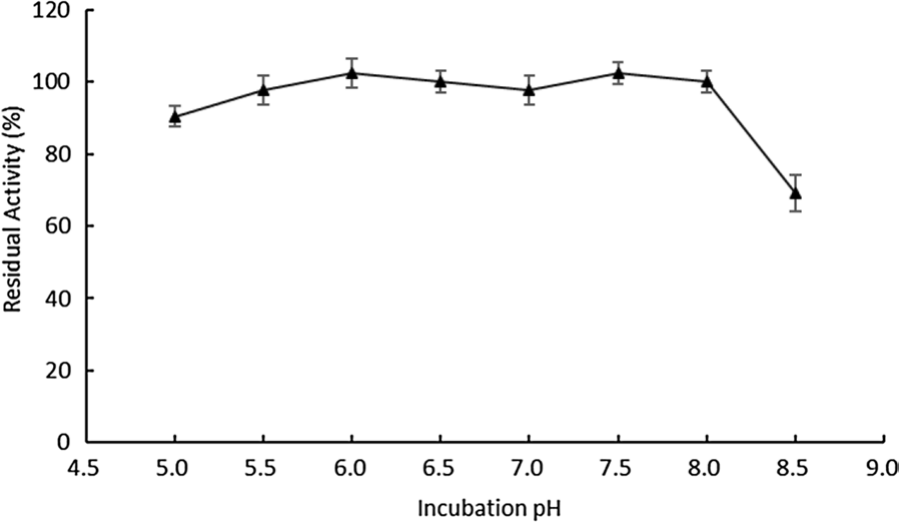
Effect of pH on the stability of recombinant OaCLH. The residual activities of recombinant OaCLH were measured with the standard chlorophyllase activity assay method, using Chl a as the substrate. Values are the means ± SD of three independent experiments

The recombinant OaCLH was stable in the pH range of 5.0–8.0. After incubation for 40 h at pH 5.0, its residual activity was 90%. After incubation for 40 h at pH 5.5, 6.0, 6.5, 7.0, 7.5, or 8.0, more than 95% of its catalytic activity was retained. However, when the incubation pH was increased to 8.5, only 69% of its catalytic activity remained (Fig. 9).

### Enzyme kinetics of recombinant OaCLH

To investigate the kinetic parameters and substrate specificity of recombinant OaCLH, the initial reaction velocities (V_0_) of full-length recombinant OaCLH toward different concentrations of four substrates (Chl a, Chl b, BChl a, and Phe a) were determined.

Table 2 summarizes the kinetic parameters of recombinant OaCLH: maximal velocity (V_max_), Michaelis constant (K_m_), catalytic activity (k_cat_), and catalytic efficiency (k_cat_/K_m_). These results show that Chl a, Chl b, BChl a, and Phe a are all reaction substrates of the recombinant OaCLH. The catalytic efficiency of the recombinant OaCLH was similar to those of chlorophyllase 1 (15.51 × 10^− 5^ s^− 1^ µM^− 1^, Chl a) and chlorophyllase 2 (0.78 × 10^− 5^ s^− 1^ µM^− 1^, Chl a) of *Brassica oleracea* [21]. Recombinant OaCLH had the highest catalytic efficiency (k_cat_/K_m_) for Chl b (6.80 × 10^− 5^ s^− 1^ µM^− 1^), followed by Chl a (3.2 × 10^− 5^ s^− 1^ µM^− 1^), and the lowest catalytic efficiency for BChl a (1.20 × 10^− 5^ s^− 1^ µM^− 1^) and Phe a (1.19 × 10^− 5^ s^− 1^ µM^− 1^). Therefore, recombinant OaCLH preferentially hydrolyzes Chl b and Chl a over BChl a and Phe a, which is consistent with the preferences of both plant and algal chlorophyllases [28, 29]. However, Chou et al. reported that recombinant CyanoCLH from the cyanobacterium *Cyanothece* sp. ATCC 51472 preferentially hydrolyzes BChl a to produce BChlide a [23]. Sharafi et al. also predicted that all bacterial and cyanobacterial chlorophyllases prefer BChl a as their substrate [24, 30]. Therefore, this is the first report of a cyanobacterial chlorophyllase with a substrate preference for Chl a and Chl b rather than BChl a. Furthermore, although recombinant OaCLH preferentially hydrolyzes Chl b and Chl a, it also shows good catalytic efficiency (k_cat_/K_m_) for BChl a and Phe a (Table 2). Therefore, recombinant OaCLH has broad substrate specificity.

**Table 2:** Substrate specificity and kinetic parameters of recombinant OaCLH

Substrate	V_max_ (10^− 3^ µmol mg^− 1^ min^− 1^)	K_m_ (µM)	K_cat_ (10^− 3^ s^− 1^)	K_cat_/K_m_ (10^− 5^ s^− 1^ µM^− 1^)
Chl a	0.98	20.41	0.65	3.20
Chl b	2.13	20.92	1.42	6.80
BChl a	16.03	888.10	10.68	1.20
Phe a	2.91	162.95	1.94	1.19

### Identification of the putative catalytic triad

A multiple sequence alignment of cyanobacterial chlorophyllases is an effective way to predict the active-site residues, because these residues are conserved well across these amino acid sequences. In this study, the amino acid sequence of OaCLH was aligned with eight previously reported cyanobacterial chlorophyllase sequences. The alignment showed that Ser159, Asp226, and His258 are strictly conserved in all cyanobacterial chlorophyllases and may therefore be the putative catalytic triad of OaCLH.

All members of the α/β hydrolase family contain a catalytic triad consisting of a nucleophile, a base, and an orientating acid [31]. Serine is responsible for the nucleophilic attack on the carbonyl carbon of the scissile ester bond. This serine lies in the consensus sequence glycine (Gly)–X–Ser–X–Gly, which is conserved in most esterases and lipases [32]. Therefore, Ser159 is a likely member of the catalytic triad of OaCLH. The catalytic histidine acts as a general base during the catalysis reactions of the α/β hydrolase family. A rule of thumb for identifying histidine as a member of the catalytic triad is that it should be located after the final central parallel β-sheet [33]. According to this rule, His258 may be one of the active-site residues that form the catalytic triad. The catalytic aspartic acid residue acts as an orientating acid and is usually located between the catalytic serine and histidine. Therefore, Asp226 may be a component of the catalytic triad.

Some site-directed mutation studies have demonstrated the importance of the catalytic triad (Ser–Asp–His) for chlorophyllase activity. Tsuchiya et al. showed that the catalytic triad (Ser162, Asp191, His262) of *Chenopodium album* chlorophyllase is essential for its enzymatic activity [17]. Lee et al. reported that three *Brassica oleracea* chlorophyllase 2 (BoCLH2) mutants, in each of which one catalytic residue (Ser141, Asp170, or His247) was replaced with alanine, had either reduced or no chlorophyllase activity [21].

We constructed the mutant enzymes S159A, D226N, and H258A with site-directed mutagenesis to identify the roles of these putative catalytic residues in OaCLH. Asp226 was substituted with asparagine (Asn) to minimize any conformational change caused by the mutation. Asp224 was also replaced with Asn to identify its function during catalysis because it is close to the putative catalytic residue Asp226.

As shown in Table 3, the mutation S159A or H258A caused the complete loss of activity, indicating that these residues are essential for the catalytic activity of OaCLH. The mutant enzyme D226N had a much lower specific activity (21% relative specific activity) than the wild-type enzyme. The D226N mutant was not completely inactivated because the catalytic Asp residue plays an auxiliary role in catalysis. The specific activity of mutant enzyme D224N was 24% higher than that of the wild-type enzyme. Therefore, Asp224 is not a catalytic residue of OaCLH. As mentioned above, the catalytic Asp residue acts as an orientating acid, and its ionized carboxyl group is required to stabilize the protonated form of the imidazole group of the catalytic His residue [34]. Because it is close to the putative catalytic residue Asp226, Asp224 is likely to interact with catalytic residue His258 and form another catalytic triad with catalytic residues Ser159 and His258. This could lead to the instability of the catalytic active center and a partial loss of activity. Asparagine (Asn) has no carboxyl group, so it cannot interact with catalytic His258 and form the catalytic triad. Therefore, the mutant enzyme D224N contains just one stable catalytic triad and showed higher specific activity than the wild-type enzyme. In summary, the results of these site-directed mutagenesis experiments suggest that the catalytic triad of OaCLH consists of Ser159, Asp226, and His258.

**Table 3:** Specific activities and relative activities of purified wild-type and mutated OaCLH. The relative specific activity of wild-type OaCLH was deemed to be 100%

Name of mutant	Specific activity (mU/mg)	Relative specific activity (%)
Wild-type	786	100
S159A	0	0
D224N	975	124
D226N	165	21
H258A	0	0

## Conclusions

A novel recombinant OaCLH was successfully expressed in *E. coli* BL21(DE3). The catalytic triad of OaCLH consists of Ser159, Asp226, and His258. The putative N-terminal 28-amino-acid signal peptide sequence of OaCLH is essential for its chlorophyllase activity, but might confer poor solubility on OaCLH. Its C-terminal fusion with a 6 × His tag caused the partial activity loss of recombinant OaCLH, but an N-terminal 6 × His tag did not destroy its activity. Recombinant OaCLH hydrolyzes Chl a, Chl b, BChl a, and Phe a, but preferentially uses Chl b and Chl a as substrates. Therefore, the strong expression and broad substrate specificity of recombinant OaCLH makes it a good candidate enzyme for genetic engineering. It may also be a promising biocatalyst for industrial production, with applications in vegetable oil refining and laundry detergents.

## Materials and methods

### Strains, plasmids and chemicals

*Escherichia coli* BL21(DE3) (Invitrogen, Carlsbad, CA, USA), and plasmids pET-24a(+) and pET-28a(+) (Novagen, Darmstadt, Germany) were used for gene expression. The Universal DNA Purification Kit and Midi Plasmid kit were purchased from Omega Bio-tek (Norcross, GA, USA) or Tiangen (Beijing, China). DNA polymerase (*KOD*) was purchased from TOYOBO (Osaka, Japan). All restriction endonucleases were purchased from New England BioLabs (Ipswich, MA, USA). T4 DNA ligase was purchased from Thermo Fisher Scientific (Waltham, MA, USA). The substrates chlorophyll a (from spinach), chlorophyll b (from spinach), and bacteriochlorophyll a (from *Rhodopseudomonas sphaeroides*) were purchased from Sigma (St. Louis, MO, USA). The substrate pheophytin a was purchased from Wako Pure Chemical industries, Ltd (Osaka, Japan).

### Sequence analysis

The putative protein sequence of *O. acuminata* chlorophyllase (OaCLH, GenBank accession no. AFY81906.1) was obtained from the NCBI database. The molecular weight, theoretical pI, estimated half-life, instability index, aliphatic index, and GRAVY value of OaCLH were predicted with the ProtParam tool (https://web.expasy.org/protparam/). The putative OaCLH sequence was also analyzed with programs TargetP-2.0 server (http://www.cbs.dtu.dk/services/TargetP/), SignalP 5.0 server (http://www.cbs.dtu.dk/services/SignalP/), and TMpred server (https://embnet.vital-it.ch/software/TMPRED_form.html) [35–38].

A multiple sequence alignment of the putative *O. acuminata* chlorophyllase and eight other published cyanobacterial chlorophyllases was constructed with Clustal Omega (https://www.ebi.ac.uk/Tools/msa/clustalo/). A homology structural model of OaCLH was constructed with the SWISS-MODEL server (https://swissmodel.expasy.org). The SAVES v5.0 server was used to verify and validate the three-dimensional structure of OaCLH (https://servicesn.mbi.ucla.edu/SAVES/).

### **Heterologous expression of OaCLH in*E. coli***

The nucleotide sequence of the *OaCLH* gene was codon optimized for *E. coli* expression. The endonuclease sites *Nde*I and *Xho*I were introduced at the 5′- and 3′-termini of the target gene, respectively, to allow the insertion of the target gene into the expression plasmids pET-24a(+) and pET-28a(+). The oligonucleotides and the codon-optimized *OaCLH* gene (full-length version and signal-peptide-sequence-truncated version) were synthesized by Shanghai Sangon Biotech Co. Ltd (China).

The synthesized DNA fragments were digested with *Nde*I and *Xho*I and ligated into pET-24a(+) and pET-28a(+) digested with the same enzymes to construct an in-frame C-terminal or N-terminal fusion protein with a 6 × His tag sequence. Competent *E. coli* BL21(DE3) cells were transformed with the resulting constructs pET-24a(+)–*OaCLH* and pET-28a(+)–*OaCLH* and the transformants were selected on Luria–Bertani (LB) medium plates containing 50 µg/mL kanamycin.

The cells carring the recombinant plasmid pET-24a(+)–*OaCLH* or pET-28a(+)–*OaCLH* were grown aerobically at 37 °C in liquid LB medium containing 50 µg/mL kanamycin until the optical density at a wavelength of 600 nm reached 0.6–0.8. The expression of the recombinant OaCLHs was then induced by the addition of IPTG at a final concentration of 0.1 mM and incubation for 16 h at 20 °C.

The recombinant-OaCLH-expressing cells were harvested by centrifugation at 10,000 × g for 10 min at 4 °C. The cell pellets were then resuspended in lysis buffer (100 mM sodium phosphate, pH 7.0) and disrupted by ultrasonication in an ice bath. After centrifugation (10,000 × g for 15 min at 4 °C), the supernatant was used as a crude extract of recombinant OaCLH. The supernatant was analyzed with chlorophyllase activity assays and SDS-PAGE. The total soluble protein content was measured with the Bradford method using bovine serum albumin as the standard [39].

### Enzyme purification

To purify of the recombinant protein, the crude enzyme was dialyzed against binding buffer (20 mM sodium phosphate, 0.5 M NaCl, 20 mM imidazole, pH 7.4) and the enzyme solution was then applied to a HisTrap HP 5 mL column (GE Healthcare, Uppsala, Sweden) previously equilibrated with binding buffer. Any unbound protein was washed out with 10 column volumes (CVs) of binding buffer. The 6 × His-tagged protein was eluted with 5 CVs of elution buffer (20 mM sodium phosphate, 0.5 M NaCl, 500 mM imidazole, pH 7.4). The enzyme fractions were pooled, concentrated with an Amicon Ultra-15 Centrifugal Filter with 10 kDa cut-off (Millipore, USA) and dialyzed against lysis buffer. The purified enzyme was analyzed with a chlorophyllase activity assay and SDS-PAGE. The protein content was measured with the Bradford method using bovine serum albumin as the standard.

### Chlorophyllase activity assay

Chlorophyllase activity was measured with a modified method described by Lee et al. [21]. The reaction mixture contained 100 µL of enzyme, 220 µL of reaction buffer (100 mM sodium phosphate, pH 7.0), 40 µL of substrate (1000 µM, dissolved in acetone), and 40 µL of acetone. The reaction mixture was incubated at 40 °C in the dark for 30 min. The reaction was then quenched by the addition of 400 µL of 10 mM KOH, 1600 µL of acetone, and 2400 µL of n-hexane. The mixture was vigorously vortexed and centrifuged at 10,000 × g for 5 min. Two separate phases were obtained. The upper n-hexane phase contained the unreacted substrate Chl a, Chl b, BChl a, or Phe a. The lower aqueous acetone phase contained the reaction product Chlide a, Chlide b, BChlide a, or Pheide a.

The amount of product was calculated from the absorbance of the aqueous acetone phase, which was measured at 665 nm for Chlide a, 650 nm for Chlide b, 773 nm for BChlide a, and 667 nm for Pheide a [28, 40]. The millimolar extinction coefficients are 54.1 mM^− 1^ cm^− 1^ for Chlide a, 42.0 mM^− 1^ cm^− 1^ for Chlide b, 42.1 mM^− 1^ cm^− 1^ for BChlide a, and 47.2 mM^− 1^ cm^− 1^ for Pheide a [28, 40]. One unit of enzyme activity was defined as the amount of enzyme required to catalyze the production of 1 nmol Chlide a, Chlide b, BChlide a, or Pheide a per minute at 40 °C. The specific activity of the enzyme was defined as the enzyme activity (units) per milligram of protein. All enzymatic assays were performed in triplicate. Control reactions, in which the enzyme was replaced with distilled water, were performed at the same temperatures, pHs, and other conditions as the chlorophyllase activity assays to ensure that there was no spontaneous substrate hydrolysis.

### Biochemical characterization of recombinant OaCLH

Purified recombinant OaCLH was biochemically characterized by measuring its hydrolysis activity against Chl a.

The optimal temperature of OaCLH for Chl a hydrolysis was determined to be 30–80 °C, according to the chlorophyllase activity assay described above. To investigate its thermal stability, enzyme samples were incubated for 60 min at temperatures ranging from 30 to 80 °C and then cooled to 4 °C. The residual activities of the enzyme samples were measured with the chlorophyllase activity assay described above.

The optimal pH was measured using 100 mM sodium acetate buffer (pH 5.0 or 5.5), 100 mM sodium phosphate buffer (pH 6.0, 6.5, 7.0, or 7.5) and 100 mM Tris-HCl buffer (pH 8.0 or 8.5). The enzyme reaction was performed with the standard assay method.

To analyze the stability of OaCLH at various pHs, enzyme solutions were dialyzed against different buffers (pH 5.0–8.5), as described above, and then incubated for 40 h at 4 °C. The residual activities were then measured with the standard assay method.

The highest activity was taken as 100%, and the percentages of the other activities were calculated relative to the highest activity. All reactions were performed in triplicate.

### Determination of enzyme kinetic parameters

Kinetic assays of purified recombinant OaCLH were performed for 30 min at 40 °C with a spectrophotometer. The initial reaction velocity (V_0_) was determined with different concentrations of substrates (10–100 µM Chl a, 10–100 µM Chl b, 50–500 µM BChl a, and 50–500 µM Phe a) in reaction buffer (100 mM sodium phosphate, pH 7.0). The production of Chlide a, Chlide b, BChlide a, and Pheide a was measured as the maximum absorbance of each product (measured at 665, 650, 773, and 667 nm, respectively). The kinetic parameters V_max_ and K_m_ were calculated with a nonlinear regression method using the Michaelis–Menten equation. All assays were performed in triplicate.

### Identification of the putative catalytic triad

The mutant enzymes S159A, D224N, D226N, and H258A were constructed to identify the role of these putative catalytic residues in the activity of OaCLH. The desired mutations were introduced into plasmid pET-28a(+)–*OaCLH* with site-directed mutagenesis using the PCR overlap extension method. The primers listed in Table 4 were designed to introduce the desired mutations. All the primers were synthesized by Shanghai Sangon Biotech Co. Ltd (China). DNA sequencing was used to confirm the veracity of the genes encoding the mutant enzymes. The recombinant strains were constructed by transforming *E. coli* BL21(DE3) cells with the pET-28a(+)–*OaCLH* derivatives, into which the desired mutations had been introduced. The culture of these transformants and the expression and purification of the mutant enzymes were performed as described above for the wild-type enzyme. The enzyme reactions were performed with the standard assay method. The protein content was measured with the Bradford method using bovine serum albumin as the standard.

**Table 4:** Oligonucleotide primers used for site-directed mutagenesis. Mutations introduced are shown in bold. *Nde*I and *Xho*I restriction sites are underlined

Primers	Oligonucleotides sequences
OaCLH-F	5’-CATATGAGCGTTATCTCTCGTCACTCTAGC-3’
OaCLH-R	5’-CTCGAGTTACTGAACGGTCTGGCCCTGAAA-3’
S159A	5’-TCTGCTGGGTCAC**GCT**TGGGGTGGCGCGG-3’
D224N	5’-GGCCTGATCGCGGGT**AAT**CGTGATGGCGTT-3’
D226N	5’-TCGCGGGTGATCGT**AAT**GGCGTTATCCAGG-3’
H258A	5’-TTCGTGGCGCTAAC**GCT**TACGGTATCACCA-3’

## Data Availability

All data generated or analysed during this study are included in this published article.

## Abbreviations

Chl: Chlorophyll
Chlase: Chlorophyllase
OaCLH: *Oscillatoria acuminata* chlorophyllase
Chl a: Chlorophyll a
Chl b: Chlorophyll b
BChl a: Bacteriochlorophyll a
Phe a: Pheophytin a
Chlide: Chlorophyllide
Chlide a: Chlorophyllide a
Chlide b: Chlorophyllide b
BChlide a: Bacteriochlorophyllide a
Pheide a: Pheophorbide a
IPTG: Isopropyl-β-D-thiogalactopyranoside
